# Supplementary material for: OGT binds a conserved C-terminal domain of TET1 to regulate TET1 activity and function in development
Source: eLife. 2018 Oct 16;7:e34870. doi: 10.7554/eLife.34870 (PMC6214653; doi:10.7554/eLife.34870)
Supplement: Supplementary file 2. [file elife-34870-supp2.docx]

| **Name** | **Purpose** | **Sequence** |
| --- | --- | --- |
| WtAmpFwd | Forward primer for amplifying Tet1 wt Gene Blocks to make HDR template | atcaaccttaacccgagaca |
| MutAmpFwd | Forward primer for amplifying Tet1 D2018A Gene Blocks to make HDR template | tcaaccttaacccgagcc |
| AmpRev | Reverse primer for amplifying Tet1 wt and D2018A Gene Blocks to make HDR template | ctttttaacagcaccggaaa |
| GenotypeFwd | Forward primer for genotyping Tet1 allele | tgatgtatcccccgaagc |
| GenotypeRev | Reverse primer for genotyping Tet1 allele | cccactacaccacattagca |
