# Supplementary material for: OGT binds a conserved C-terminal domain of TET1 to regulate TET1 activity and function in development
Source: eLife. 2018 Oct 16;7:e34870. doi: 10.7554/eLife.34870 (PMC6214653; doi:10.7554/eLife.34870)
Supplement: Supplementary file 3. [file elife-34870-supp3.docx]

| **Name** | **Sequence** |
| --- | --- |
| Tet1 wt-3xF-T2A-GFP | gcagaccgggagtgtcctgatgtatcccccgaagccaatttatcacaccaaattccttctcgagttgcatcaaccttaacccgagacaatgttgttaccgtgtccccatactctctcactcatgttgcgggaccatacaatcgttgggtcgactacaaagaccatgacggtgattataaagatcatgatatcgattacaaggatgacgatgacaagggaagcggagagggcagaggaagtctgctaacatgcggtgacgtcgaggagaatcctggacctgtgagcaagggcgaggagctgttcaccggggtggtgcccatcctggtcgagctggacggcgacgtaaacggccacaagttcagcgtgtccggcgagggcgagggcgatgccacctacggcaagctgaccctgaaatttatttgcacgacagggaagctgcccgtgccctggcccaccctcgttacgaccctaacatatggcgtgcagtgcttcagccgctacccggatcatatgaagcaacacgacttctttaagtcagccatgcccgaaggctacgtccaggagcgcaccatcttcttcaaggacgacggcaactacaagacccgcgccgaggtgaagttcgagggcgacaccctggtgaaccgcatcgagctgaagggcatcgacttcaaggaggacggcaacatcctggggcacaagctggagtacaactacaacagccacaacgtctatatcatggccgacaagcagaagaacggcatcaaggtgaacttcaagatccgccacaacatcgaggacggcagcgtgcagctcgccgaccactaccagcagaacacccccatcggcgacggccccgtgctgctgcccgacaaccactacctgagcacccagtccgccctgagcaaagaccccaacgagaagcgcgatcacatggtcctgctggagttcgtgaccgccgccgggatcactctcggcatggacgagctgtacaagtaaaagcttctctcatgtaatgcatttgctaatgtggtgtagtgggtatttttgtttgtttgtttgttttcttttgtttttttgttttttccggtgctgttaaaaagaaagtcattctgttgtttactgtagctttgtttcgcccatttc |
| Tet1 D2018A-3xF-T2A-GFP | gcagaccgggagtgtcctgatgtatcccccgaagccaatttatcacaccaaattccttctcgagttgcatcaaccttaacccgagccaatgttgttaccgtgtccccatactctctcactcatgttgcgggaccatacaatcgttgggtcgactacaaagaccatgacggtgattataaagatcatgatatcgattacaaggatgacgatgacaagggaagcggagagggcagaggaagtctgctaacatgcggtgacgtcgaggagaatcctggacctgtgagcaagggcgaggagctgttcaccggggtggtgcccatcctggtcgagctggacggcgacgtaaacggccacaagttcagcgtgtccggcgagggcgagggcgatgccacctacggcaagctgaccctgaaatttatttgcacgacagggaagctgcccgtgccctggcccaccctcgttacgaccctaacatatggcgtgcagtgcttcagccgctacccggatcatatgaagcaacacgacttctttaagtcagccatgcccgaaggctacgtccaggagcgcaccatcttcttcaaggacgacggcaactacaagacccgcgccgaggtgaagttcgagggcgacaccctggtgaaccgcatcgagctgaagggcatcgacttcaaggaggacggcaacatcctggggcacaagctggagtacaactacaacagccacaacgtctatatcatggccgacaagcagaagaacggcatcaaggtgaacttcaagatccgccacaacatcgaggacggcagcgtgcagctcgccgaccactaccagcagaacacccccatcggcgacggccccgtgctgctgcccgacaaccactacctgagcacccagtccgccctgagcaaagaccccaacgagaagcgcgatcacatggtcctgctggagttcgtgaccgccgccgggatcactctcggcatggacgagctgtacaagtaaaagcttctctcatgtaatgcatttgctaatgtggtgtagtgggtatttttgtttgtttgtttgttttcttttgtttttttgttttttccggtgctgttaaaaagaaagtcattctgttgtttactgtagctttgtttcgcccatttc |
| Tet1 wt-3xF-T2A-tdTomato | gcagaccgggagtgtcctgatgtatcccccgaagccaatttatcacaccaaattccttctcgagttgcatcaaccttaacccgagacaatgttgttaccgtgtccccatactctctcactcatgttgcgggaccatacaatcgttgggtcgactacaaagaccatgacggtgattataaagatcatgatatcgattacaaggatgacgatgacaagggaagcggagagggcagaggaagtctgctaacatgcggtgacgtcgaggagaatcctggacctgtttccaaaggggaggaagtcattaaggaatttatgaggttcaaagtgcgcatggagggatctatgaacggccacgaatttgagatagaaggcgaaggcgagggaaggccctacgagggcactcagactgctaagctgaaagtaactaagggtggtcctctgcctttcgcctgggatatcctgtcaccccagtttatgtacggtagtaaagcttatgtgaagcatcccgctgatatacctgactataaaaaactgtccttcccagagggcttcaagtgggagcgagtaatgaactttgaagatggtggactggttaccgttacccaagattcatctttgcaggacggaacattgatctacaaggtcaagatgcggggcactaacttcccacccgacgggccagtcatgcagaagaagactatgggctgggaagctagtactgagcgactctaccctagagatggtgtcttgaaaggggagattcatcaagcactgaaattgaaagacggcggtcattacctcgtcgaattcaaaaccatatacatggccaaaaagcctgtgcaactgccagggtattattatgtcgacacaaaactcgatataaccagccataatgaagattataccatagtcgaacaatatgaacgctctgaaggacgacatcatttgttcttgggacatgggactggatccacaggatccggttcctctggaacagcatcctccgaagacaataatatggccgtaataaaagaattcatgcgattcaaagtgagaatggaaggaagtatgaatggtcacgagtttgaaatcgagggagaaggagagggtcggccctatgagggtacacagacagctaagttgaaggttactaagggcggccctcttccctttgcttgggatattctctccccacaattcatgtacgggtccaaggcttacgtaaaacatcccgctgatatacccgattacaaaaaactgtccttccccgaaggctttaaatgggaaagggtgatgaatttcgaggacgggggattggtaactgtcacacaggattcctctcttcaagatggaacactgatttacaaggtaaaaatgagagggaccaactttccccctgatgggcccgtgatgcaaaagaaaaccatgggctgggaagcatctaccgagagactttatcccagggacggcgttcttaagggagagattcaccaagctttgaaacttaaggatggaggtcactacctcgtggagtttaagacaatatatatggcaaaaaaaccagtccaactccccggatactattacgttgataccaaactggacataacttctcataacgaggactacactatagtggaacaatatgaacgctctgagggtcgacaccaccttttcctgtatggaatggatgaactgtataagtagtaaaagcttctctcatgtaatgcatttgctaatgtggtgtagtgggtatttttgtttgtttgtttgttttcttttgtttttttgttttttccggtgctgttaaaaagaaagtcattctgttgtttactgtagctttgtttcgcccatttc |
| Tet1 D2018A-3xF-T2A-tdTomato | gcagaccgggagtgtcctgatgtatcccccgaagccaatttatcacaccaaattccttctcgagttgcatcaaccttaacccgagccaatgttgttaccgtgtccccatactctctcactcatgttgcgggaccatacaatcgttgggtcgactacaaagaccatgacggtgattataaagatcatgatatcgattacaaggatgacgatgacaagggaagcggagagggcagaggaagtctgctaacatgcggtgacgtcgaggagaatcctggacctgtttccaaaggggaggaagtcattaaggaatttatgaggttcaaagtgcgcatggagggatctatgaacggccacgaatttgagatagaaggcgaaggcgagggaaggccctacgagggcactcagactgctaagctgaaagtaactaagggtggtcctctgcctttcgcctgggatatcctgtcaccccagtttatgtacggtagtaaagcttatgtgaagcatcccgctgatatacctgactataaaaaactgtccttcccagagggcttcaagtgggagcgagtaatgaactttgaagatggtggactggttaccgttacccaagattcatctttgcaggacggaacattgatctacaaggtcaagatgcggggcactaacttcccacccgacgggccagtcatgcagaagaagactatgggctgggaagctagtactgagcgactctaccctagagatggtgtcttgaaaggggagattcatcaagcactgaaattgaaagacggcggtcattacctcgtcgaattcaaaaccatatacatggccaaaaagcctgtgcaactgccagggtattattatgtcgacacaaaactcgatataaccagccataatgaagattataccatagtcgaacaatatgaacgctctgaaggacgacatcatttgttcttgggacatgggactggatccacaggatccggttcctctggaacagcatcctccgaagacaataatatggccgtaataaaagaattcatgcgattcaaagtgagaatggaaggaagtatgaatggtcacgagtttgaaatcgagggagaaggagagggtcggccctatgagggtacacagacagctaagttgaaggttactaagggcggccctcttccctttgcttgggatattctctccccacaattcatgtacgggtccaaggcttacgtaaaacatcccgctgatatacccgattacaaaaaactgtccttccccgaaggctttaaatgggaaagggtgatgaatttcgaggacgggggattggtaactgtcacacaggattcctctcttcaagatggaacactgatttacaaggtaaaaatgagagggaccaactttccccctgatgggcccgtgatgcaaaagaaaaccatgggctgggaagcatctaccgagagactttatcccagggacggcgttcttaagggagagattcaccaagctttgaaacttaaggatggaggtcactacctcgtggagtttaagacaatatatatggcaaaaaaaccagtccaactccccggatactattacgttgataccaaactggacataacttctcataacgaggactacactatagtggaacaatatgaacgctctgagggtcgacaccaccttttcctgtatggaatggatgaactgtataagtagtaaaagcttctctcatgtaatgcatttgctaatgtggtgtagtgggtatttttgtttgtttgtttgttttcttttgtttttttgttttttccggtgctgttaaaaagaaagtcattctgttgtttactgtagctttgtttcgcccatttc |
