# Supplementary material for: OGT binds a conserved C-terminal domain of TET1 to regulate TET1 activity and function in development
Source: eLife. 2018 Oct 16;7:e34870. doi: 10.7554/eLife.34870 (PMC6214653; doi:10.7554/eLife.34870)
Supplement: Supplementary file 4. [file elife-34870-supp4.docx]

| **Gene** | **Primers** |
| --- | --- |
| *Peg10* | Fwd: gaatcctcgtgtggaacag |
|  | Rev: cagttggaggaaccaccc |
| *Slc38a4* | Fwd: gccaaggaaggagggtctc |
|  | Rev: ggctccaatgttctgcattg |
| *Lefty1* | Fwd: ctatggagctcaaggcaatt |
|  | Rev: gttctaggatccagttctcg |
| *Lefty2* | Fwd: ggttcctgacgtatgaatgt |
|  | Rev: ctccttcacactgacaatca |
| *Tet2* | Fwd: gtcaacaggacatgatccaggag |
|  | Rev: cctgttccatcaggcttgct |
