# Supplementary material for: OGT binds a conserved C-terminal domain of TET1 to regulate TET1 activity and function in development
Source: eLife. 2018 Oct 16;7:e34870. doi: 10.7554/eLife.34870 (PMC6214653; doi:10.7554/eLife.34870)
Supplement: Supplementary file 5. — (A) Representative images of larvae with high and low runx1 expression. (B) Embryo numbers and scoring for all five biological replicates. [file elife-34870-supp5.pptx]

## Slide 1
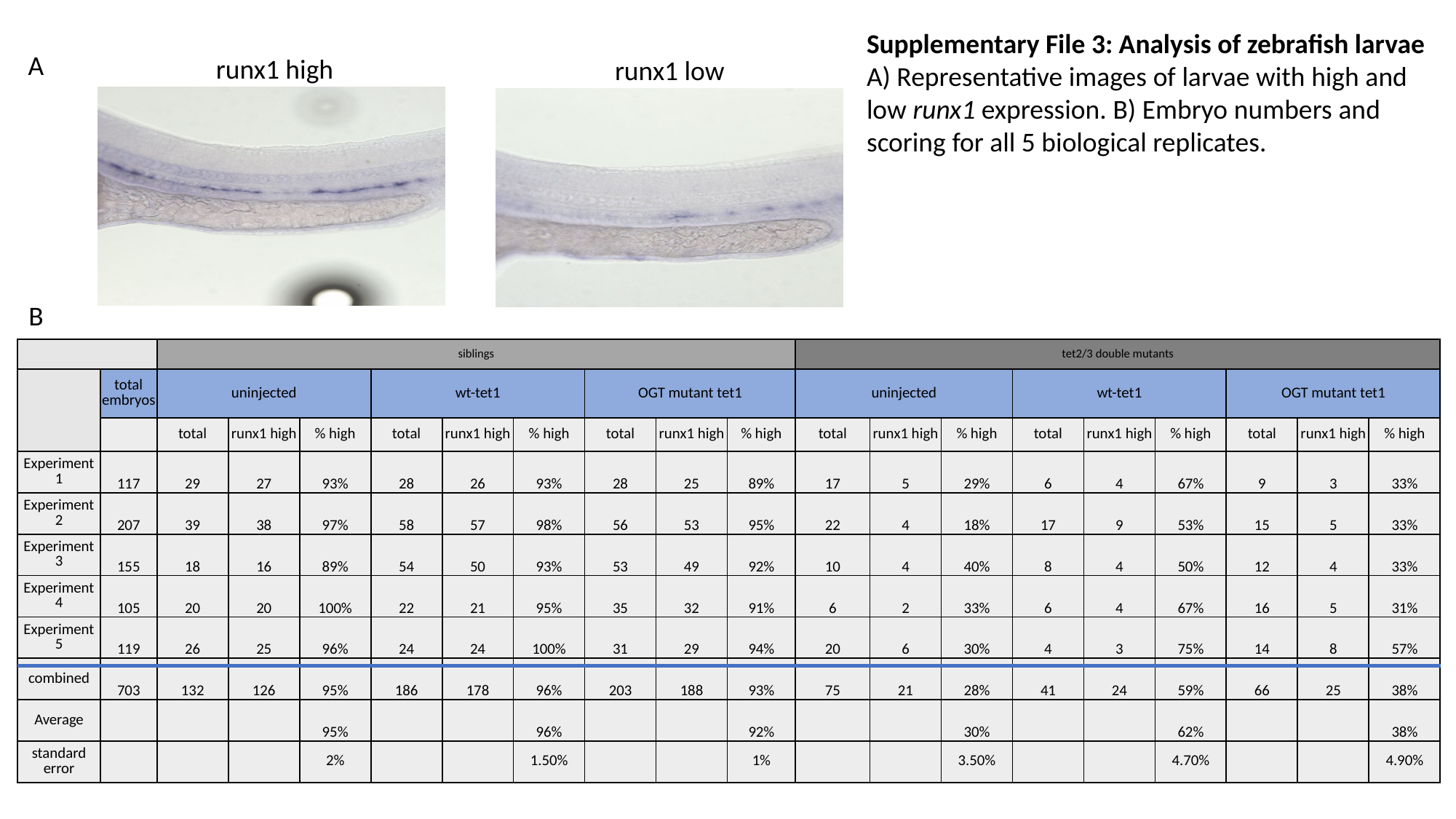

Supplementary File 3: Analysis of zebrafish larvae
A) Representative images of larvae with high and low runx1 expression. B) Embryo numbers and scoring for all 5 biological replicates.
A
runx1 high
runx1 low
B
| | | siblings | | | | | | | | | tet2/3 double mutants | | | | | | | | |
| --- | --- | --- | --- | --- | --- | --- | --- | --- | --- | --- | --- | --- | --- | --- | --- | --- | --- | --- | --- |
| | total embryos | uninjected | | | wt-tet1 | | | OGT mutant tet1 | | | uninjected | | | wt-tet1 | | | OGT mutant tet1 | | |
| | | total | runx1 high | % high | total | runx1 high | % high | total | runx1 high | % high | total | runx1 high | % high | total | runx1 high | % high | total | runx1 high | % high |
| Experiment 1 | 117 | 29 | 27 | 93% | 28 | 26 | 93% | 28 | 25 | 89% | 17 | 5 | 29% | 6 | 4 | 67% | 9 | 3 | 33% |
| Experiment 2 | 207 | 39 | 38 | 97% | 58 | 57 | 98% | 56 | 53 | 95% | 22 | 4 | 18% | 17 | 9 | 53% | 15 | 5 | 33% |
| Experiment 3 | 155 | 18 | 16 | 89% | 54 | 50 | 93% | 53 | 49 | 92% | 10 | 4 | 40% | 8 | 4 | 50% | 12 | 4 | 33% |
| Experiment 4 | 105 | 20 | 20 | 100% | 22 | 21 | 95% | 35 | 32 | 91% | 6 | 2 | 33% | 6 | 4 | 67% | 16 | 5 | 31% |
| Experiment 5 | 119 | 26 | 25 | 96% | 24 | 24 | 100% | 31 | 29 | 94% | 20 | 6 | 30% | 4 | 3 | 75% | 14 | 8 | 57% |
| combined | 703 | 132 | 126 | 95% | 186 | 178 | 96% | 203 | 188 | 93% | 75 | 21 | 28% | 41 | 24 | 59% | 66 | 25 | 38% |
| Average | | | | 95% | | | 96% | | | 92% | | | 30% | | | 62% | | | 38% |
| standard error | | | | 2% | | | 1.50% | | | 1% | | | 3.50% | | | 4.70% | | | 4.90% |
